# The Effects of Marine Fungal Asterripeptides A–C on In Vitro and In Vivo *Staphylococcus aureus* Skin Infection

**DOI:** 10.3390/ph17101345

**Published:** 2024-10-08

**Authors:** Ekaterina A. Chingizova, Ekaterina A. Yurchenko, Artur R. Chingizov, Anna A. Klimovich, Evgeny A. Pislyagin, Ekaterina S. Menchinskaya, Aleksandra S. Kuzmich, Phan Thi Hoai Trinh, Ngo Thi Duy Ngoc, Tran Thi Thanh Van, Irina V. Guzhova, Dmitry L. Aminin, Anton N. Yurchenko

**Affiliations:** 1 G.B. Elyakov Pacific Institute of Bioorganic Chemistry, Far Eastern Branch of the Russian Academy of Sciences, Pr. 100-letya Vladivostoka 159, 690022 Vladivostok, Russia; chingizova_ea@piboc.dvo.ru (E.A.C.); pislyagin@hotmail.com (E.A.P.); yurchenkoan@piboc.dvo.ru (A.N.Y.); 2 Nhatrang Institute of Technology Research and Application, Vietnam Academy of Science and Technology, Nha Trang 650000, Vietnam; phanhoaitrinh84@gmail.com (P.T.H.T.);; 3 Institute of Cytology, Russian Academy of Sciences, Tikhoretsky Ave., 4, 194064 St. Petersburg, Russia; irina.guzhova@incras.ru; 4 Department of Biomedical Science and Environmental Biology, Kaohsiung Medical University, Kaohsiung 80708, Taiwan

**Keywords:** marine fungi, secondary metabolites, antibiotic, biofilms, skin infection, *Staphylococcus aureus*, antioxidant, wound healing

## Abstract

**Objectives:** This study aimed to investigate the in vitro and in vivo antibacterial and cytoprotective activities of marine fungal tripeptide derivatives with cinnamic acid moiety asterripeptides A–C (**1**–**3**). **Methods:** The antimicrobial and antibiofilm activities of asterripeptides A–C were tested using the *Staphylococcus aureus* ATCC 21027 strain. Human HaCaT keratinocytes infected with *S. aureus* were used for the in vitro investigation of the various aspects of the influence of asterripeptides A–C by lumino- and fluorospectrometry, ELISA, flow cytometry, Western blotting, and microscopy techniques. In the in vivo experiments, mice with burns and scalped *S. aureus*-infected wounds were used according to ethical committee resolution. **Results:** Asterripeptides A–C (10 µM) inhibited *S. aureus* growth and biofilm formation. Asterripeptides A–C increased the viability, proliferation, and migration of *S. aureus*-infected HaCaT cells and reduced the release of reactive oxygen species (ROS), NO, TNF-α, and IL-18. Asterripeptides A–C protected HaCaT cells against TNF-α-induced inflammation, decreased the transcriptional level of NF-κB in JB6 Cl41 cells, and increased the protein levels of Nrf2 and glutathione synthetase in HaCaT cells. More active asterripeptide C was tested in in vivo burn wounds and *S. aureus*-infected incised wounds. Asterripeptide C significantly enhanced wound healing, normalized cytokine levels and profiles of peripheral blood samples, and decreased *S. aureus* contamination of wounds and blood in mice with infected incised wounds. **Conclusions:** Taken together, these results confirm the dual antibacterial and Nrf2-dependent anti-inflammatory activities of asterripeptides A-C in in vitro and in vivo assays.

## 1. Introduction

In accordance with the Global Action Plan on Antimicrobial Resistance from the World Health Organization [1], the actual strategy of antibiotic discovery involves the search for substances aimed not only at suppressing growth by inhibiting bacterial ribosomal protein synthesis or nucleic acid synthesis, as modern antibiotics do, but also at inhibiting virulence and biofilm formation, as well as involving protective cellular systems to resist infections [2]. Inhibitors of biofilm formation are particularly important given that biofilm forms of bacterial infection significantly prolong the healing of infected wounds or complicate the treatment of bronchopulmonary infections [3]. In April 2024, a new synthetic thiodiazinone drug, “Fluorothiazinone”, was registered, which is capable of specifically suppressing motility, toxin secretion, invasion, colonization, and biofilm formation by pathogenic bacteria *Acinetobacter baumannii* and *Pseudomonas aeruginosa* [4,5]. Nevertheless, the continuation of research and the search for new drugs that not only suppress bacterial functions but also activate human intracellular defense systems remain relevant.

Marine fungi are a known source of secondary metabolites with various bioactivities, including antibacterial activities. Competitive interactions between fungi and bacteria play an important role in microbial communities, which provokes microorganisms to produce exolites that control the growth and development of their competitors. From 1998 to 2019, 272 antimicrobial compounds were isolated from marine fungi [6]. Most of these compounds have been tested for their ability to inhibit growth of pathogenic bacteria. The effect of about a dozen-and-a-half marine fungal metabolites on the formation of bacterial biofilms has been described [7].

As of 2019, approximately 131 peptides had been reported from 17 genera of marine fungi [8], and nearly 53% of these exhibited cytotoxic, antimicrobial, and antiviral activities. In addition to affecting bacterial growth, peptides can inhibit biofilm formation, such as *cis*-cyclo (Leucyl-Tyrosyl) from sponge-derived *Penicillium* sp. F37 [9]. A review of the literature published from January 1991 to June 2023 described 366 marine fungal non-ribosomal (NRPs) and ribosomally synthesized and post-translationally modified (RiPP) peptides belonging to linear, cyclic, and depsi classes, one-fifth of which showed antimicrobial and antibiofilm properties [10]. Cyclohexapeptide enniatines have been isolated from various fungi and reported to destroy bacterial cell walls via ionophoric action [11]. The drug fusafungin, consisting of a complex of enniathins, has been actively used to treat upper respiratory tract infections; however, its use was banned by the European Medical Agency in 2016 because of rare but severe allergic reactions in the form of bronchospasm [12]. However, enniatin B has recently been shown to inhibit the formation of *Candida albicans* biofilms [13], which opens up new possibilities for its therapeutic use. The non-ribosomal lipopeptide pneumocandin B_0_ from *Glarea lozoyensis* fungus became the basis for the first class of caspofungin acetate (CANCIDAS^®^), a parenteral antifungal drug [14]. However, the potential of non-ribosomal fungal peptides is not limited to this, and further research may reveal new promising molecules. 

**Figure 1:**
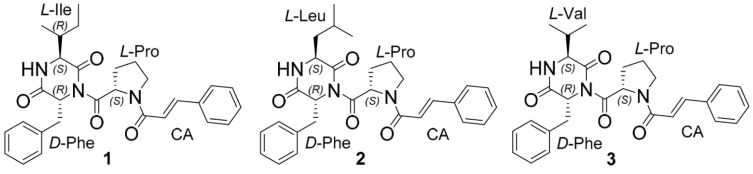
The chemical structures of asterripeptide A (**1**), asterripeptide B (**2**), and asterripeptide C (**3**).

Recently, new cyclopeptides with unusual cinnamic acid moiety asterripeptides A–C (**1**–**3**) (Figure 1) from the mangrove-derived fungus *Aspergillus terreus* LM5.2 were isolated by our research team [15]. It was reported that **2** and **3** significantly inhibited the activity of staphylococcal sortase A enzyme in a cell-free test. 

The aims of this work were to study various aspects of the in vitro antibacterial and cytoprotective activities of asterripeptides A–C (**1**–**3**) and to investigate the most active compound in in vivo skin wound experiments.

## 2. Results

### 2.1. Antibacterial Activity of Asterripeptides A–C

The effect of **1**–**3** on *Staphylococcus aureus* growth and biofilm formation is shown in Table 1. All the compounds showed weak antimicrobial activity and a significant reduction in biofilm formation. The compounds **1**–**3** at 100 µM inhibited *S. aureus* growth by 57.33%, 49.79%, and 35.3%, respectively. At a concentration of 25 µM, compound **3** inhibited *S. aureus* growth by only 13.56% (Table 1). 

**Figure 2:**
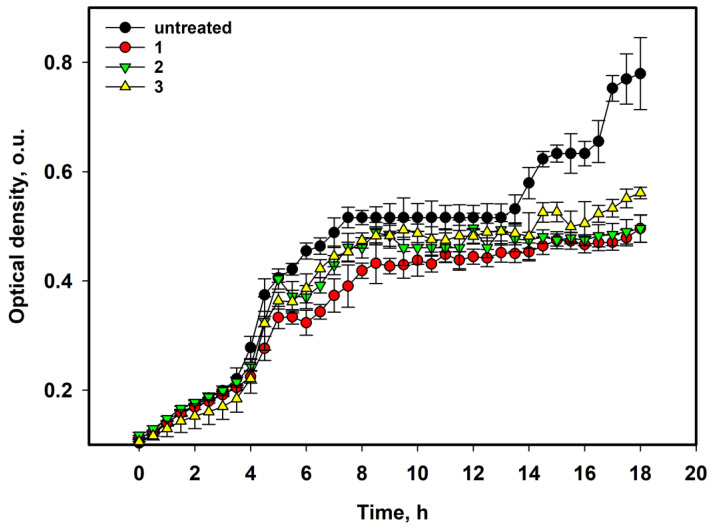
The influence of asterripeptides A–C (**1**–**3**) on *S. aureus* suspension growth for 18 h. In the graph legend, the numbers **1**–**3** correspond to investigated compounds: **1** is asterripeptide A, **2** is asterripeptide B, **3** is asterripeptide C. The concentration of each compound was 50 µM. All experiments were performed in triplicate. Data are presented as the mean ± SEM.

The growth curves are shown in Figure 2. A decrease in the bacterial suspension density was observed after 4–5 h of treatment with the compounds at a concentration of 50 µM, and some bacteriostatic influence was detected after 14 h (Figure 2).

**Figure 3:**
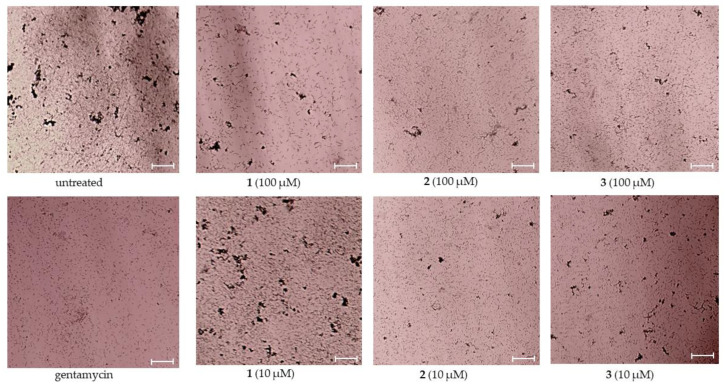
Visualization of MTT-straining *Staphylococcus aureus* biofilms after treatment with asterripeptides A–C (**1**–**3**) for 18 h. Scale bar is 500 µm. In the figure captions, the numbers **1**–**3** correspond to investigated compounds: **1** is asterripeptide A, **2** is asterripeptide B, **3** is asterripeptide C.

Moreover, the effect of **1**–**3** on *S. aureus* biofilm formation was investigated, and the data are presented in Table 1. Compounds **1**–**3** inhibited the bacterial biofilm formation by nearly half at concentrations of both 100 and 10 µM (Table 1). The biofilms stained with 3-(4,5-dimethyltiazol-2-yl)-2,5-diphenyl-tetrazolium bromide (MTT) were visualized by light microscopy and are presented in Figure 3.

Thus, **1**–**3** significantly inhibited the formation of *S. aureus* biofilms and prevented the growth of *S. aureus* cultures. Next, we investigated the effects of **1**–**3** at 10 µM in HaCaT keratinocytes co-cultured with *S. aureus* as an in vitro model of skin infection.

### 2.2. The Effect on the Viability of S. aureus-Infected HaCaT Keratinocytes

**Figure 4:**
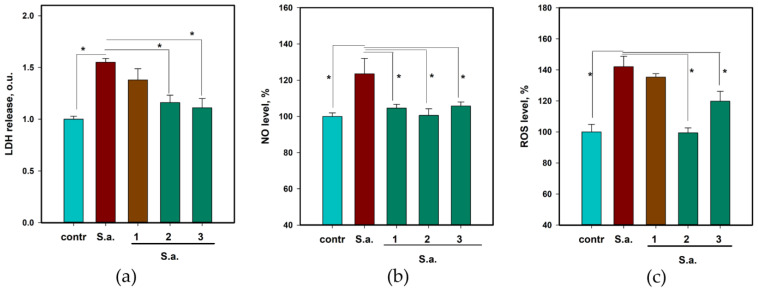
The influence of asterripeptides A–C (**1**–**3**) on lactate dehydrogenase (LDH) release (**a**), NO level (**b**), and ROS formation (**c**) in *S. aureus*-infected HaCaT cells. In the *X*-axis captions, numbers **1**–**3** correspond to investigated compounds: **1** is asterripeptide A, **2** is asterripeptide B, **3** is asterripeptide C. The LDH release was measured after 24 h. The NO and ROS levels were measured after 3 h. All compounds were used at a concentration of 10 µM. All experiments were performed in triplicate, and data are presented as the mean ± standard error of the mean (SEM). Asterisk * indicates the significance of the difference with a *p*-value < 0.05.

**Figure 5:**
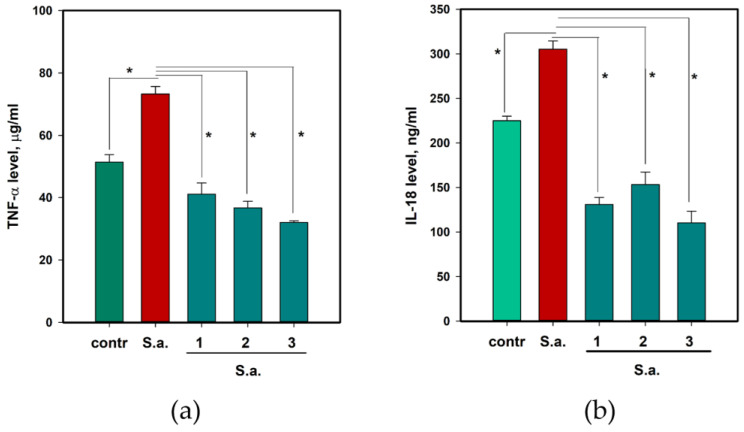
The influence of asterripeptides A-C (**1**–**3**) on cytokines TNF-α (**a**) and IL-18 (**b**) release in *S. aureus*-infected HaCaT cells. In the *X*-axis captions, the numbers **1**–**3** correspond to investigated compounds: **1** is asterripeptide A, **2** is asterripeptide B, **3** is asterripeptide C. Cytokine release was measured for 24 h. All the compounds were used at a concentration of 10 µM. All experiments were performed in triplicate. The data are presented as the mean ± SEM. Asterisk * indicates the significance of the difference with a *p*-value < 0.05.

To determine the influence of **1**–**3** on *S. aureus*-infected HaCaT keratinocytes, lactate dehydrogenase (LDH) release, reactive oxygen species (ROS), and nitrogen (II) oxide (NO) levels, tumor necrosis factor α (TNF-α) and interleukin-18 (IL-18) cytokine levels were measured. The data are presented in Figure 4 and Figure 5, respectively.

Compounds **1**–**3** up to 100 µM were non-toxic to HaCaT cells. Incubation of HaCaT cells with *S. aureus* led to a significant deterioration in the viability of keratinocytes and an increase in the release of the LDH enzyme into the extracellular medium by 53.4% (Figure 4a). Compounds **2** and **3** at a concentration of 10 µM decreased the LDH release by 20.2% and 22.3%, respectively. Asterripeptide A (**1**) also decreased LDH release, but the difference was not statistically significant.

*S. aureus* infection increased the NO levels in HaCaT cells by 23.5% after 3 h (Figure 4b). Compounds **1**–**3** reduced this increase by 15.3%, 18.5%, and 14.3%, respectively. ROS levels in *S. aureus*-infected HaCaT cells increased by 42.1%, whereas compounds **2** and **3** decreased ROS by 30.0% and 15.7%, respectively (Figure 4c).

*S. aureus* caused the increase in both TNF-α (Figure 5a) and IL-18 (Figure 5b) levels and all compounds reduced this increase. Infection with *S. aureus* induced TNF-α release by 42.5% and **1**–**3** decreased it by 43.9%, 44.9%, and 56.3%, respectively. IL-18 levels increased by 35.7% and **1**–**3** decreased it by 56.9%, 49.8%, and 63.9%, respectively.

Thus, asterripeptides A–C (**1**–**3**) protect HaCaT cells against *S. aureus*-induced damage, oxidative stress, and inflammation. 

### 2.3. The Effect of ***1***–***3*** on the Proliferation of S. aureus-Infected HaCaT Keratinocytes

**Figure 6:**
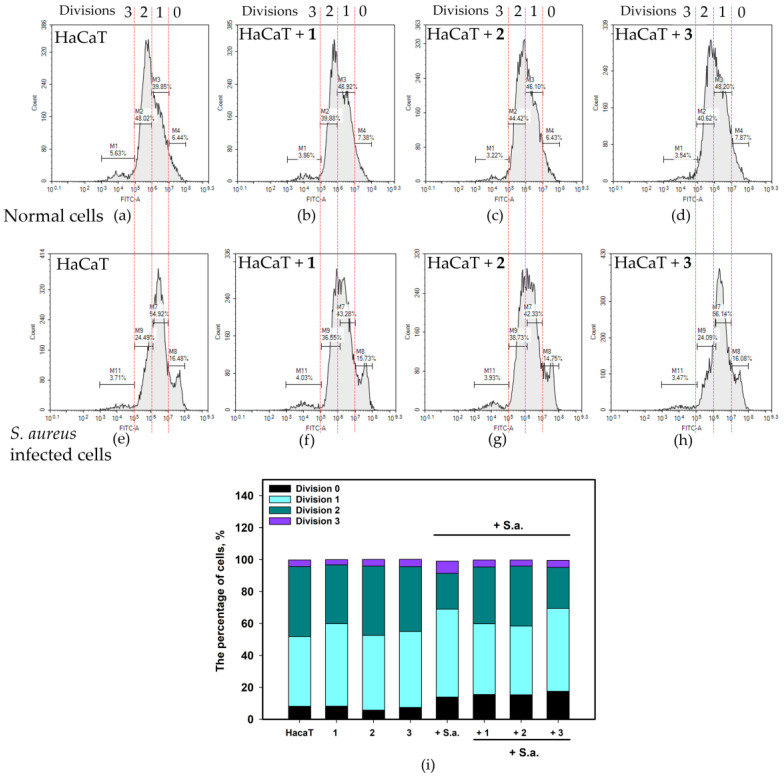
The influence of asterripeptides A–C (**1**–**3**) on *S. aureus*-infected HaCaT cell proliferation. In the figures and *X*-axis captions, the numbers **1**–**3** correspond to investigated compounds: **1** is asterripeptide A, **2** is asterripeptide B, **3** is asterripeptide C. (**a**) Non-infected HaCaT cells; (**b**–**d**) non-infected HaCaT cells treated with **1**, **2**, and **3**, respectively; (**e**) *S. aureus*-infected cells; (**f**–**h**) *S. aureus*-infected cells treated with **1**, **2**, and **3**, respectively. Percentage of cells in each cell-cycle phase (**i**). The compounds were used at concentrations of 10 µM.

The proliferation profile of HaCaT cells was studied using carboxyfluorescein diacetate succinimidyl ester (CFDA SE) fluorescence dye and flow cytometry. The results are shown in Figure 6.

In non-infected HaCaT cells, the percentage of division 0 cells was 8.1%, that of division 1 cells was 43.7%, that of division 2 cells was 43.9%, and that of division 3 cells was 4.1% (Figure 6). Compounds **2** and **3**, as well as compound **1** (*p* value = 0.158), had no significant effect on the HaCaT proliferation profile. *S. aureus* infection significantly inhibited HaCaT proliferation, as the percentage of cells in divisions 0 and 1 increased to 13.9% and 55.1%, respectively, and the percentage of cells in division 2 decreased to 22.4%.

Asterripeptides A (**1**) and B (**2**) reduced the percentage of cells in division 1 and increased the percentage of cells in division 2, whereas asterripeptide C (**3**) decreased the percentage of cells in division 2 by increasing the percentage of cells in division 3. These results indicated a recovery of the proliferation in *S. aureus*-infected HaCaT cells as a result of the influence of **1**–**3**.

### 2.4. The Effect of ***1***–***3*** on Cell Cycle of S. aureus-Infected HaCaT Keratinocytes

**Figure 7:**
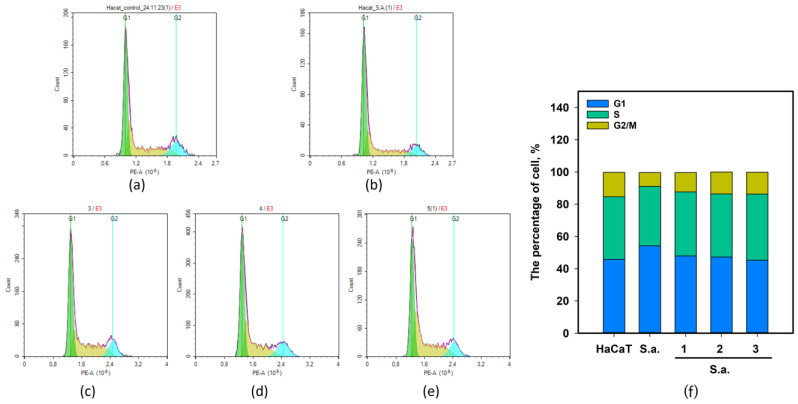
The influence of asterripeptides A–C (**1**–**3**) on *S. aureus*-infected HaCaT cell cycle. In the *X*-axis captions, the numbers **1**–**3** correspond to investigated compounds: **1** is asterripeptide A, **2** is asterripeptide B, **3** is asterripeptide C. (**a**) Non-infected HaCaT cells; (**b**) *S. aureus*-infected cells; (**c**–**e**) *S. aureus*-infected cells treated with **1**–**3**, respectively. (**f**) Percentage of cells in each cell-cycle phase. The compounds were used at concentrations of 10 µM.

The effect of **1**–**3** on the cell cycle of *S. aureus*-infected HaCaT keratinocytes was investigated by flow cytometry. The data are presented in Figure 7.

As previously reported [16], infection with *S. aureus* arrested the HaCaT cell cycle in the G1 phase. The percentage of *S. aureus*-infected cells in the G1 phase was 54.2 ± 1.6%, while the percentage of non-infected cells in the G1 phase was 45.8 ± 2.8%. The percentage of *S. aureus*-infected cells in the G2/M phase was 8.7 ± 1.1%, while the percentage of non-infected cells in the G2/M phase was 15.2 ± 1.4%. Compounds **1**–**3** induced the normalization of the HaCaT cell cycle (Figure 7). The percentage of cells in the G1 phase was 47.9%, 47.3%, and 45.2% when *S. aureus*-infected cells were treated with **1**–**3**, respectively. Thereafter, the percentage of cells in the S phase was 39.7%, 39.2%, and 41.1%, and in the G2/M phase, it was 12.1%, 13.6%, and 13.7%, respectively.

Thus, asterripeptides A-C (**1**–**3**) could restore cell-cycle progression and proliferation in *S. aureus*-infected keratinocytes.

### 2.5. The Effect of ***1***–***3*** on Migration of HaCaT Keratinocytes in In Vitro Model of S. aureus-Infected Skin Wound

**Figure 8:**
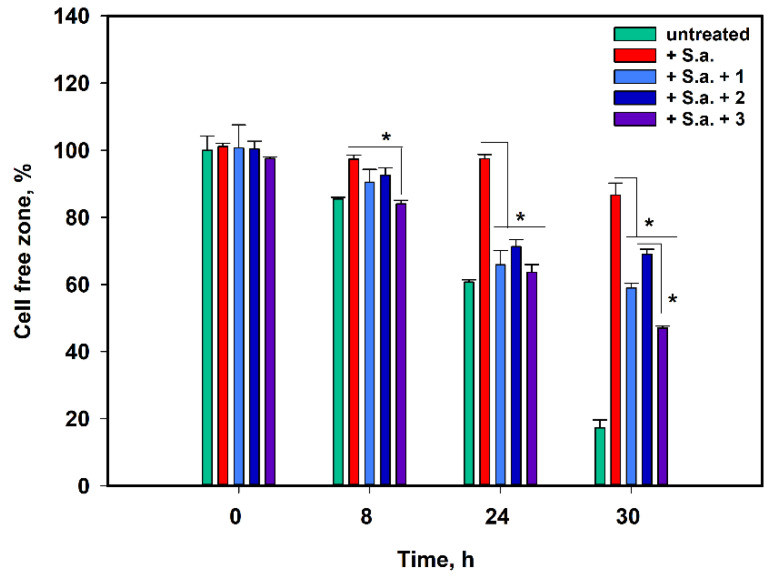
The effect of asterripeptides A-C (**1**–**3**) on the migration of HaCaT cells in in vitro skin wounds infected with *S. aureus*. In the graph legend, the numbers **1**–**3** correspond to investigated compounds: **1** is asterripeptide A, **2** is asterripeptide B, **3** is asterripeptide C, S.a. is *Staphylococcus aureus*. The compounds were used at concentrations of 10 µM. All experiments were performed in triplicate. The data are presented as the mean ± SEM. Asterisk * indicates the significance of the difference with a *p*-value < 0.05.

The effect of **1**–**3** on HaCaT migration in in vitro skin infected wounds was investigated using a scratch assay. The data are presented in Figure 8.

The cell-free zone in the untreated HaCaT cell monolayer was filled by 15% after 8 h, 40% after 24 h, and 83% after 30 h (Figure 8). Compounds **1**–**3** did not affect the migration of untreated HaCaT cells. *S. aureus* infection caused dramatic stagnation of keratinocyte migration in the cell-free zone because the proximity of the cell-free zone was only 13% after 30 h. The effects of compound **3** were observed after the first 8 h of the experiment. After 30 h of observation, compounds **1**–**3** induced the close of the cell-free zone by 41%, 31%, and 53%, respectively. Notably, compound **3** was more effective than compounds **1** and **2**.

Thus, asterripeptides A–C (**1**–**3**) can protect keratinocytes against *S. aureus*-induced damage, oxidative stress, and inflammation, and recover the intensity of proliferation and migration in in vitro experiments. To clarify the anti-inflammatory and antioxidant activities of **1**–**3**, we investigated the effects of **1**–**3** on the transcriptional activity of NF-κB in JB6 Cl41 cells, as well as on NO production and viability in TNF-α-treated HaCaT cells. The levels of nuclear factor erythroid 2-related factor 2 (Nrf2) and glutathione synthetase (GSS) were measured in HaCaT cells.

### 2.6. The Anti-Inflammatory and Antioxidant Activity of ***1***–***3***

**Figure 9:**
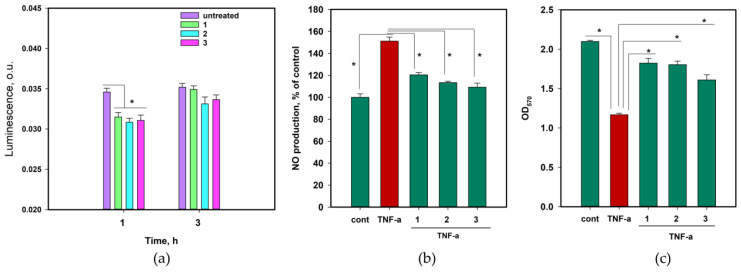
The influence of asterripeptides A–C (**1**–**3**) on the luminescence in JB6-Luc NF-κB cells (**a**). The influence of **1**–**3** on NO production (**b**) and viability (**c**) of TNF-α-treated HaCaT cells. In the graph legend (**a**) and *X*-axis captions (**b**,**c**), the numbers **1**–**3** correspond to investigated compounds: **1** is asterripeptide A, **2** is asterripeptide B, **3** is asterripeptide C. NO levels were measured after 3 h. Formazan production was measured after 24 h. All the compounds were used at a concentration of 10 µM. All experiments were carried out in triplicate, and data are presented as the mean ± standard error of mean (SEM). Asterisk * indicates the significance of the difference with a *p*-value < 0.05.

The effects of compounds **1**–**3** on the transcriptional activity of NF-κB were examined in JB6 Cl41 cells stably expressing a luciferase reporter gene controlled by NF-κB [17]. The data are shown in Figure 9a. 

The compounds **1**–**3** after 1 h reduced the luciferase activity in JB6-Luc NF-κB cells by 8.9%, 10.7%, and 10.1%, respectively. After 3 h, the effects of compounds **2** and **3** were reductions of 5.9% and 4.3%, respectively, with p-values of 0.102 and 0.108, respectively.

The anti-inflammatory effects of **1**–**3** were determined via experiments, then HaCaT cells were treated with recombinant TNF-α. The production of NO and viability of TNF-α-treated cells were measured, and the data are presented in Figure 9b,c.

Treatment of HaCaT cells with TNF-α for 3 h increased NO production by 51.2% (Figure 9b). Compounds **1**–**3** decreased NO level in TNF-α-induced HaCaT cells by 20.4%, 25.0%, and 27.8%, respectively. In addition, treatment of HaCaT cells with TNF-α decreased the production of formazan and its viability by 44.3% (Figure 9c). Compounds **1**–**3** increased the viability of TNF-α treated HaCaT cells by 56.2%, 54.4%, and 37.7%, respectively.

**Figure 10:**
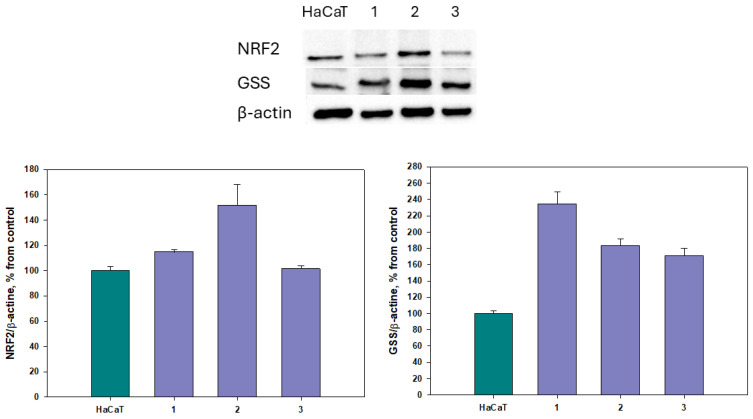
The influence of asterripeptides A–C (**1**–**3**) on the on Nrf2 and GSS levels in HaCaT cells. In the *X*-axis captions, the numbers **1**–**3** correspond to investigated compounds: **1** is asterripeptide A, **2** is asterripeptide B, **3** is asterripeptide C. Protein levels were measured after 24 h. All the compounds were used at a concentration of 10 µM. All experiments were carried out in triplicate, and data are presented as the mean ± standard error of mean (SEM).

The effect of **1**–**3** on the levels of phosphorylated Nrf2 and GSS in HaCaT cells was measured using specific antibodies and Western blotting. b-Actin was used as a background. The data are presented in Figure 10.

The level of GSS increased by 148%, 91%, and 61%, respectively, when HaCaT cells were treated with asterripeptides A–C (**1**–**3**) for 24 h. Moreover, compounds **1** and **2** increased the level of phosphorylated Nrf2 by 16% and 78%, respectively; however, it cannot be excluded that this would be observable for all compounds at different time points.

Thus, **1**–**3** can downregulate the transcriptional activity of NF-κB and reduce TNF-α-mediated inflammation. Moreover, asterripeptides A–C (**1**–**3**) had a pronounced effect on the cellular antioxidant machinery.

Molecular docking of **1**–**3** with the Kelch domain of Keap1 (PDB ID 1U6D) was performed, and the description of the obtained interactions is presented in Table 2.

**Figure 11:**
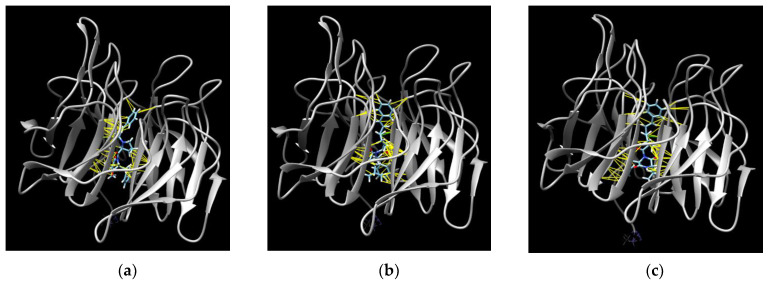
The molecular docking of Kelch domain of Keap1 (PDB ID 1U6D) (white) with asterripeptide A (**a**), asterripeptide B (**b**), and asterripeptide C (**c**) (blue). The red color indicates oxygen atoms in the compound structures. The green lines show the predicted hydrogen bonds between the compounds and Keap1, and the yellow lines show hydrophobic interactions.

Previously, the building site of the Nrf2 peptide with the Kelch domain was calculated with Arg415, Arg483, Ser439, Arg459, Hsd436, and Thr481 amino acid residues (hydrogen binding) and hydrophobic interactions with Tyr525, Ala556, Tyr572, Tyr334, Cys434, ILe435, ILe461, Gly462, Phe478, Gly480, and Thr481 [18]. In these calculations, all predicted complexes were located in the central channel of the Kelch domain (Figure 11) and all compounds formed hydrophobic interactions between Ala556 and the cinnamic moiety. This can prevent interactions between the Kelch domain of Keap1 and Nrf2 peptide. Moreover, hydrophobic interactions were calculated with Cys513 (for **2**) or Cys515 and Cys368 (for **1** and **3**), similar to sulforaphane, a known covalent inhibitor of Keap1 activity and activator of the Keap1/Nrf2-dependent antioxidant pathway [19].

### 2.7. The Effect of Asterripeptide C in In Vivo Experiments

All investigated compounds showed significant inhibition of *S. aureus* biofilm formation and cytoprotective activity, and we chose asterripeptide C (**3**) for in vivo experiments. Similar to compounds **1** and **2**, asterripeptide C (**3**) significantly inhibited *S. aureus* biofilm formation. However, its inhibitory activity against *S. aureus* growth was greater than that of compounds **1** and **2**. Moreover, asterripeptide C (**3**) showed greater cytoprotective effects in *S. aureus*-infected HaCaT cells. Therefore, we considered asterripeptide C (**3**) as the leading peptide and used it in in vivo assays.

Burn wounds and *S. aureus*-infected incised wounds in mice were prepared, and the effects of compound **3** on the wound area, blood cell profile, and blood cytokine levels were studied. Asterripeptide C (**3**) was dissolved in a small amount of dimethyl sulfoxide (DMSO) (100 µL) and then dissolved in carboxymethylcellulose (CMC) hydrogel [20,21]; for example, the concentration of **3** was 0.5 mkg per g of hydrogel. CMC hydrogel with DMSO without the compound was used as the base control. Levomekol was used as a reference drug. Detailed descriptions of mouse treatments are provided in the Section 4.13.

#### 2.7.1. The Effect of Asterripeptide C (**3**) against Burn Wounds

**Figure 12:**
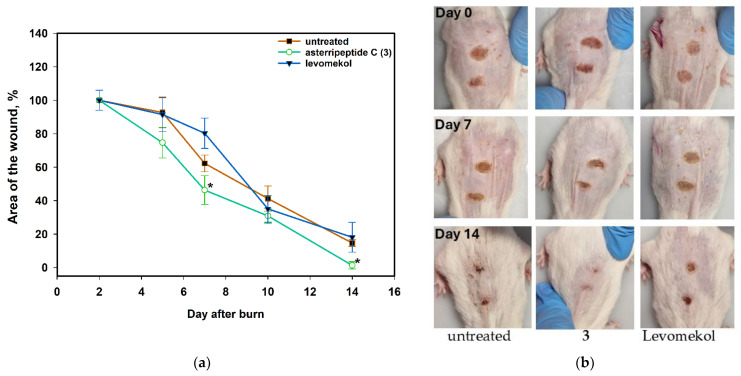
The effect of asterripeptide C (**3**) on burn wound healing in mice (n = 6). Area of the wound during healing (**a**) and visualization on days 7 and 14 (**b**). The data are presented as the mean ± standard error of the mean (SEM). Asterisk * indicates significant differences (*p* < 0.05) in wound healing between mice treated with asterripeptide C (**3**) and untreated mice.

Briefly, the mice were anesthetized for the burn wound, which was inflicted with a red-hot metal rod. The medication was started the next day and administered daily. The wound area was measured the next day and then after 3–4 days. The data are presented in Figure 12.

CMC hydrogels without an active compound did not enhance wound healing. Application of asterripeptide C (**3**) to burn wounds accelerates wound healing. On day 5, the area of the untreated wound decreased by 7.3%, while the area of the wound treated with **3** decreased by 25.4%. On day 10, the untreated wound area was reduced by 58.8%, whereas the area of the wound treated with **3** was reduced by 70%, and this enhancement of wound healing was significant. On the last day of observation, the area of the wound treated with compound **3** healed completely, whereas the untreated wound area was 14.6% of the initial area.

After the experiment was completed, the mice were euthanized and peripheral blood samples were obtained and analyzed. The data are presented in Table 3.

Burn wounds in mice were accompanied by a two-fold increase in neutrophil content in peripheral blood samples because the neutrophil concentration was 1.703 × 10^9^/L in intact mice and 3.615 × 10^9^/L in untreated mice with burn wounds (*p* value = 0.049). The application of asterripeptide C (**3**) to burn wounds normalized the leukocyte profile of the blood samples and decreased the level of neutrophils to 1.377 × 10^9^/L (*p* = 0.034). In addition, a visible increase in platelet concentration of 46% was detected in blood samples from untreated mice with burn wounds, although this change was not significant (*p* = 0.162), and the application of **3** decreased it to an intact level (*p* = 0.043).

Notably, the base (CMC hydrogel) and levomekol had no effect on the peripheral blood profiles of mice with burn wounds.

#### 2.7.2. The Effect of Asterripeptide C (**3**) against *S. aureus*-Infected Incised Wound

**Figure 13:**
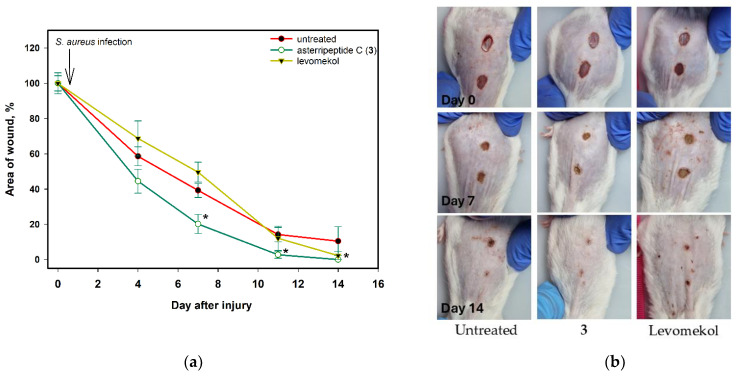
The effect of asterripeptide C (**3**) on *S. aureus*-infected incised wound healing in mice (n = 6). The area of the wound during healing (**a**) and visualization on days 7 and 14 (**b**). The data are presented as the mean ± standard error of mean (SEM). Asterisk * indicates the significant differences (*p* value < 0.05) of wound healing in mice treated with asterripeptide C (**3**) in comparison with untreated mice.

To prepare the *S. aureus*-infected incised wound in mice, the animals were anesthetized, and the wound was inflicted using a scalpel for skin biopsy. After 2 h, the suspension of *S. aureus* was sterile and applied to the wound. The medication was started the next day and was administered daily. The wound area was measured before infection and then after each 3–4 days. The data are presented in Figure 13.

The CMC hydrogel without the active compound did not enhance wound filing. Asterripeptide C (**3**) ensured complete healing after 11 days, while the area of untreated incised wounds was 19% of that of the original wounds (Figure 13). On day 4, the area of untreated wounds decreased by 41.4%, while the application of **3** reduced the wound area by 55.6%. On day 7, the area of untreated wounds was reduced by 60.8%, while the area of wounds treated with **3** was reduced by 79.9%.

The peripheral blood profile was measured on days 4 and 12. Blood samples were obtained from previously euthanized mice. The results are presented in Table 4.

On day 4, a significant increase in neutrophil concentration in peripheral blood samples from mice with *S. aureus*-infected incised wounds to 2.798 × 10^9^/L, in contrast with intact mice, with 1.290 × 10^9^ of neutrophils per liter, was observed (*p* value = 0.048). Treatment with **3** slightly decreased the neutrophil concentration to 1.953 × 10^9^/L (*p* value = 0.095) in blood samples. Additionally, a significant increase in platelet concentration was detected in the blood samples of mice with *S. aureus*-infected incised wounds (*p* value = 0.023). Asterripeptide C (**3**) caused a weak decrease in platelet concentration (*p* = 0.111).

On day 12, the blood profile of untreated mice with *S. aureus*-infected incised wounds changed dramatically. The concentrations of leukocytes (WBC) were reduced by half (*p* = 0.187), neutrophils by 67.5% (*p* = 0.120), and lymphocytes by 69.2% (*p* = 0.305) in untreated mice compared with those in intact animals. The concentration of hemoglobin (HGB) in mice with infected wounds was significantly reduced to 86.333 g/L, whereas it was 142.667 g/L in the blood of intact mice (*p* = 0.007). The application of asterripeptide C (**3**) ensured that the blood profile approached an intact level. HGB in the blood of treated mice was 155.000 g/L (*p* = 0.051), and the leukocyte formula was close to normal (Table 4).

Despite the fact that most changes in blood profiles were not significant (*p* > 0.05), it is important to note the negative effects of *S. aureus* infection on mouse peripheral blood and the positive effects of asterripeptide C (**3**).

**Figure 14:**
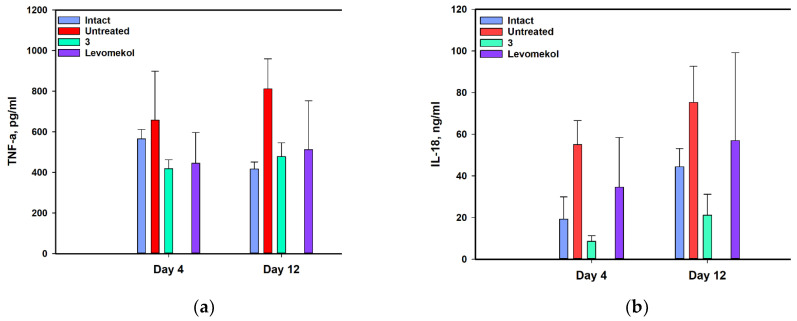
The influence of asterripeptide C (**3**) on the on the TNF-α (**a**) and IL-18 (**b**) levels in peripheral blood of mice with *S. aureus*-infected incised wounds. All tests were conducted in triplicate. The data are presented as the mean ± SEM.

Cytokine levels significantly increased in mice infected with *S. aureus* (Figure 14). *S. aureus* infection increased TNF-α levels by 16.3% (*p* = 0.727) and 94.7% (*p* = 0.0611) on days 4 and 12, respectively. Asterripeptide C (**3**) reduced TNF-α levels by 36.4% (*p* = 0.384) on day 4 and 41.0% (*p* = 0.111) on day 12. 

*S. aureus* infection increased IL-18 levels by 187.1% (*p* = 0.0846) and 69.4% (*p* = 0.187) on days 5 and 12, respectively. Asterripeptide C (**3**) reduced IL-18 levels by 84.4% (*p* = 0.0169) on day 4 and 71.9% (*p* = 0.0542) on day 12.

**Figure 15:**
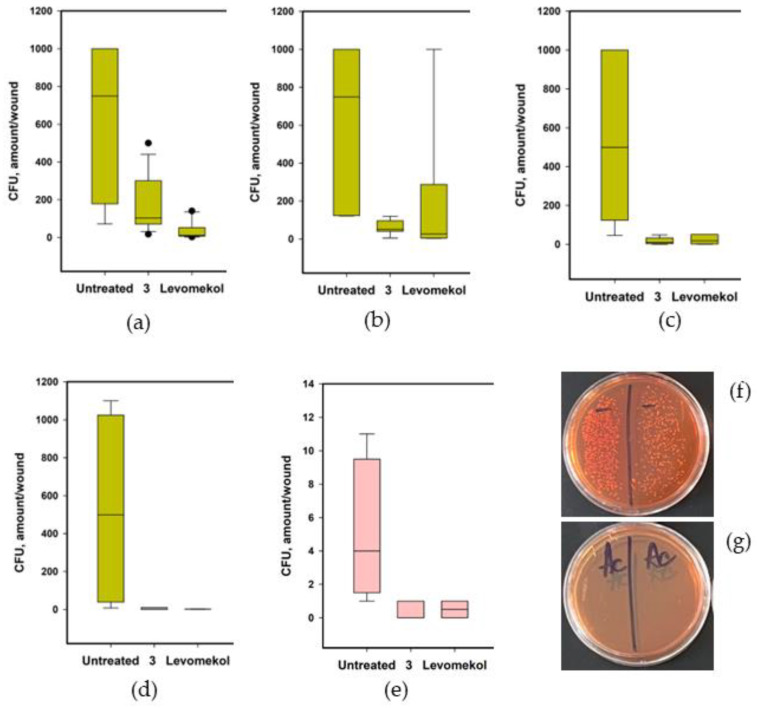
The effect of **3** on *S. aureus* contamination of the incised wounds on days 3 (**a**), 5 (**b**), 9 (**c**), and 12 (**d**); and *S. aureus* contamination of blood samples (**e**). Representative images of *S. aureus* colonies on Petri dishes with samples from untreated mice wounds (**f**) and wounds treated with **3** (**g**).

Bacterial growth in *S. aureus*-infected incised wounds was also studied. The data are presented in Figure 15. A large number of bacterial colonies were isolated from untreated incised wounds infected with *S. aureus* in all experiments (Figure 15f). Moreover, *S. aureus* colonies (1–11 per sample) were isolated from the blood samples of untreated mice at the end of the experiment (Figure 15e). Asterripeptide C (**3**) rapidly decreased wound contamination. The reference drug levomekol also inhibited contamination of incised wounds, while some increase in contamination was detected on day 5. Moreover, only one *S. aureus* colony was isolated from all the blood samples (n = 6) obtained from mice treated with **3**.

## 3. Discussion

In the present study, we found that marine fungal tripeptides with cinnamic acid moiety, asterripeptides A–C (**1**–**3**), can inhibit the growth of *Staphylococcus aureus* and prevent the formation of bacterial biofilms. Moreover, the antibacterial effect of asterripeptide C (**3**) was confirmed in an in vivo experiment, in which *S. aureus* contamination of wounds in mice was reduced. Equally importantly, these compounds exhibit Keap1/Nrf2-dependent antioxidant and anti-inflammatory properties. These dual activities of **1**–**3** resulted in the protection of HaCaT keratinocytes against *S. aureus*-induced damage, which was confirmed in in vivo experiments. Thus, asterripeptides A–C in in vitro assays and asterripeptide C in in vivo assays were found to be promising antibacterial and cytoprotective molecules.

The presence of the cinnamic acid moiety in the structure of asterripeptides is crucial for their biological activity. Cinnamic acid is a natural aromatic carboxylic acid widely observed in various plants [22]. The first step of this pathway is catalyzed by phenylalanine ammonia lyase, which is found in plants but can also be found in fungi because their encoding genes have been discovered in various ascomycetes and basidiomycetes [23]. However, cinnamic acid derivatives have been isolated much less frequently from fungi than plants. Therefore, methyl 2-{(E)-2-[4-(formyloxy)phenyl]ethenyl}-4-methyl-3-oxopentanoate was obtained from *Pyronema* sp. isolated from *Taxus mairei* [24]. ^14^C labeling helps in concluding that fungi metabolize cinnamate (obtained from phenylalanine) to benzoate [25], which can then be used for the biosynthesis of various bioactive molecules, including asperphenamate [26]. Such a deep biosynthetic change in cinnamic acid explains the rare isolation of its neighboring derivatives from fungi. This is all the more interesting when they are isolated, since cinnamic acid derivatives have a wide range of biological activities, including antimicrobial [27] and cytoprotective [28] activities, due to their significant role in the adaptation of fungus to stress via their influence on AP1 stress response transcription factor [29]. Chlorogenic acid, which is an ester formed between quinic acid and *trans*-cinnamic acid, can inhibit the activity of staphylococcal sortase A (SrtA) enzyme, and prolong survival and protect mice from early death in *S. aureus* infection [30]. Salvianolic acid A inhibits SrtA activity and protects mice against lethal pneumonia caused by MRSA in combination with latamoxef [31]. In both cases, the carbonyl group of cinnamic acid was found to be significant for interaction with Arg197 in the active site of SrtA. Hydroxylated cinnamic acid derivatives have antioxidant, anti-inflammatory, and ultraviolet (UV) protective effects on skin [32]. Finally, the imidazole para-substituted cinnamic acid, known as ozagrel, has been employed therapeutically for treating acute ischemic stroke [33] and as an anti-aggregate molecule [34].

Some hydroxylated derivatives of cinnamic acid have been reported to induce the Keap1/Nrf2 pathway in human cells [35], and we found similar effects for asterripeptides A–C. In addition, the peptide chain in asterripeptides A–C also ensures the presence of antioxidant and anti-inflammatory activities. Our calculations showed that Ile, Leu, and Val in the structures of **1**–**3**, respectively, can interact with cysteine residues in the Kelch domain of Keap1. This led to activation of the Keap1/Nrf2/ARE antioxidant pathway, which was confirmed by measuring Nrf2 and glutathione synthetase levels. Nrf2 is a transcription factor that is downregulated by Keap1 in the cytosol and is translocated to the nucleus when its interaction with Keap1 is disrupted. In the nucleus, Nrf2 activates ARE genes, initiates the expression of various antioxidant enzymes, and suppresses the NF-κB-dependent inflammatory pathway [36]. Glutathione synthetase (GSS) catalyzed the second step of the synthesis of glutathione [37], which is one of the most antioxidant in eukaryotes. Earlier, Nrf2-dependent anti-inflammatory effects have been reported for other marine fungal metabolites, asperpropanols A–D [38], and we now propose this activity for asterripeptides A–C.

The dual antibacterial and cytoprotective effects of asterripeptides A–C were found in in vitro experiments, and these effects were confirmed in in vivo skin wound assays. This compound enhanced the healing of burn wounds and normalized the peripheral blood profiles in these mice. Additionally, it significantly enhanced the healing of incised wounds infected with *S. aureus* and normalized the peripheral blood profile, as well as the inflammatory TNF-α and IL-18 cytokine levels.

Skin and soft tissue infections sometimes become an entryway for *S. aureus* into the deeper tissues and bloodstream (*S. aureus* bacteremia), which can result in sepsis with a paradoxical immunosuppressive response together with inflammation [39]. The disruption of neutrophils and, to a lesser extent, of monocytes and macrophages as a result of the action of a staphylococcal bicomponent pore-forming toxin, Panton-Valentine Leukocidin, has been reported in some invasive infections [40], and we observed this in our investigation, together with a decrease in the hemoglobin level. However, the application of asterripeptide C (**3**) reduced *S. aureus* contamination of the wound and prevented bacteria from entering the bloodstream, as well as the disruption of neutrophils.

In our in vivo experiments, the ointment levomekol with a combination of chloramphenicol (7.5 mg/g) and dioxomethyltetrahydropyrimidin (40 mg/g) was used as a reference drug [41]. This ointment is recommended for the treatment of infected skin wounds as both an antibiotic and regenerative drug. In the present study, asterripeptide C at a significantly lower concentration of 0.5 mg per g of CMC hydrogel showed an anti-contaminant effect like that of levomekol; however, its anti-inflammatory effect and wound healing velocity was more expressed. 

Thus, cyclic non-ribosomal peptides with an unusual fragment of cinnamic acid asterripeptides A–C are promising tools for the treatment of infected wounds in comparison with commonly used antibiotics. Future research should focus on their stability, bioavailability, and safety when administered internally, as well as their effectiveness against clinical bacterial isolates, including antibiotic-resistant isolates. It is also necessary to further investigate ways of obtaining them in sufficient quantities: will their synthesis be effective, or will we need a super-producing fungal strain?

## 4. Materials and Methods

### 4.1. Compounds

Asterripeptides A–C (**1**–**3**) were isolated from the culture media of the mangrove-derived fungus *Aspergillus terreus* LM5.2. The strain was stored in the Collection of Marine Microorganisms of G.B. Elyakov Pacific Institute of Bioorganic Chemistry (Vladivostok, Russia) and Collection of Marine Microorganisms of the Nha Trang Institute of Technology Research and Application VAST (Nha Trang, Vietnam). The isolation and chemical structure elucidation of **1**–**3** have been previously reported in detail [15]. Prior to the start of the experiments, the compounds were stored dry at −20 °C. After dissolution in DMSO (10 mM), the compounds were immediately used at a concentration range of 1–100 µM. The concentration of DMSO used was 1% or less.

### 4.2. Bacterial Strain and Antimicrobial Assays

*Staphylococcus aureus* ATCC 21027 was fermented in a Petri dish at 37 °C for 24 h on solid Mueller Hinton broth medium with agar (16.0 g/L).

The antimicrobial activity of the compounds was tested at concentrations up to 100 µM, according to [42]. Gentamicin was used as a positive control at a concentration of 1 mg/mL, and 1% DMSO solution in phosphate-buffered saline (PBS) was used as a negative control. The optical density of the bacterial suspension after 18 h was measured at a 620 nm wavelength.

The effect of compounds on the biofilm formation for 18 h was tested using MTT reagent (Sigma-Aldrich, St. Louis, MO, USA), according to [43]. The optical density of the obtained solution was measured at a 570 nm wavelength. A MultiskanFS spectrophotometer (Thermo Scientific Inc., Beverly, MA, USA) was used in both assays. The results were calculated as percentages of the control data.

### 4.3. Cell Lines and Culture Conditions

The human HaCaT keratinocyte cell line was kindly provided by Prof. N. Fusenig (Cancer Research Center, Heidelberg, Germany). The cells were incubated in DMEM medium (BioloT, St. Petersburg, Russia) containing 10% fetal bovine serum (FBS) and 1% penicillin/streptomycin (BioloT, St. Petersburg, Russia) in humidified 5% CO_2_ at 37 °C. Dimethyl sulfoxide solution (DMSO) in PBS was used as a negative control, and the concentration of DMSO was not more than 1%.

The murine epidermal cell line JB6 P+ Cl41 and its stable transfectants JB6-Luc NF-κB cells were cultured in MEM with 5% of FBS, 2 mM of L-glutamine, and 1% of penicillin/streptomycin at 37 °C and 5% CO_2_. The transformed cells were selected using GeneCitine G-418 (Sigma-Aldrich, St. Louis, MO, USA).

### 4.4. The Infection of HaCaT Cells with Staphyloccocus aureus

HaCaT cells (1.2 × 10^4^ cells/well) were seeded in 96-well plates and incubated for 24 h. A suspension of *S. aureus* (10^2^ CFU/mL) in full DMEM was added to each well instead of the culture medium. DMEM without the *S. aureus* suspension was added to the control wells. Compounds at a concentration of 10 μM were added to the wells after 1 h, and *S. aureus*-infected HaCaT cells were cultured for 48 h.

### 4.5. Cell Viability Assays

#### 4.5.1. Lactate Dehydrogenase (LDH) Release Test

An LDH Cytotoxicity Assay Kit (Abcam, Cambridge, UK) was used to measure LDH release from the cells. The absorbance of the reaction mixture was measured at λ = 450 nm using a Multiskan FC microplate photometer (Thermo Scientific Inc., Beverly, MA, USA) and expressed in optical units (o.u.).

#### 4.5.2. Formazan Production (MTT) Assay

MTT (3-(4,5-dimethylthiazol-2-yl)-2,5-diphenyltetrazolium bromide) from Sigma-Aldrich (St. Louis, MO, USA) was used to assess the cell viability. The absorbance of the formed formazan was measured at λ = 570 nm using a Multiskan FC microplate photometer (Thermo Scientific Inc., Beverly, MA, USA) and expressed in optical units (o.u.).

### 4.6. Cytokines, ROS and NO Level Measure

The infected *S. aureus* cells (Section 4.4) were treated with the compounds (10 µM) for 48 h, and then the samples were prepared as described previously [44]. TNF-α levels in the mixture of supernatants and cell lysates were immediately analyzed using the Human TNF-α ELISA kit (SEA133Hu), and IL-18 levels were analyzed using the Human IL-18 ELISA kit (SEA064Hu) from Cloud-Clone (Houston, TX, USA).

For ROS detection, the infected cells were treated with compounds (10 µM) for 3 h and the specific fluorescence dye 2,7-dichlorofluorescein diacetate (Sigma-Aldrich, St. Louis, MO, USA), at a concentration of 10 μM. The fluorescence of 2,7-dichlorofluorescein was detected at λ_ex_ = 485 and λ_em_ = 520 nm. For NO detection, the specific fluorescence dye diaminofluorescein-FM diacetate (Sigma-Aldrich, St. Louis, MO, USA) at a concentration of 10 μM was used after treating infected cells with compounds (10 µM) for 3 h. The intensity of the diaminofluorescein-FM fluorescence was measured at λ_ex_ = 485 and λ_em_ = 520 nm.

A PHERAstar FS plate reader (BMG Labtech, Offenburg, Germany) was used for all experiments. The data were processed using MARS Data Analysis v. 3.01R2 (BMG Labtech, Offenburg, Germany).

### 4.7. TNF-α-Induced Inflammation in HaCaT Cells

HaCaT cells (1.2 × 10^4^ cells/well) were seeded in 96-well plates for 24 h. Human recombinant tumor necrosis factor α (TNF-α) (Sci-Store, Skolkovo, Russia) was added at a concentration of 10 pg/mL to the HaCaT cell culture, and the compound was added after 1 h. The NO level was measured after 3 h, as described in Section 4.6.

### 4.8. Migration of S. aureus-Infected HaCaT Cells

Silicon 2-well inserts (Ibidi^®^, Gräfelfing, Germany) were used to prepare cell-free zones in the HaCaT cell monolayer. After removing the inserts, the cells were labeled with (5,6)-carboxyfluorescein succinimidyl ester (CFDA SE) dye (LumiTrace CFDA SE kit; Lumiprobe, Moscow, Russia). Then, the *S. aureus* suspension (10^2^ CFU/mL) in full DMEM was added as necessary. A medium without bacteria was added to the control wells. The compound (10 μM) was added to the wells after 1 h and HaCaT and *S. aureus* cells were cultured at 37 °C in a humidified atmosphere with 5% (*v*/*v*) CO_2_. HaCaT cell migration was observed using an MBF-10 fluorescence microscope (Lomo Microsystems, St. Petersburg, Russia). The ImageJ 1.53t software (Rasband W.S., NIH, Bethesda, MD, USA) was used to calculate the filling of the cell-free zone with HaCaT cells.

### 4.9. Flow Cytometry

#### 4.9.1. Proliferation of *S. aureus*-Infected HaCaT Cells

HaCaT cells at a concentration of 1.2 × 10^4^ were seeded in a 12-well plate for 24 h. After adhesion, the cells were stained with (5,6)-carboxyfluorescein succinimidyl ester (CFDA SE) dye (LumiTrace CFDA SE kit; Lumiprobe, Moscow, Russia). An *S. aureus* suspension (10^2^ CFU/mL) in full DMEM was added to the wells, and after 1 h, the compound (10 µM) was added to the wells. Medium without bacterial suspension was added to the control wells.

After 48 h of incubation, cells were washed twice with PBS, centrifuged, and collected. The intensity of CFDA fluorescence was analyzed using a NovoCyte flow cytometer (Agilent, Austin, TX, USA) and the data were analyzed as previously described [45].

#### 4.9.2. Cell Cycle of *S. aureus*-Infected HaCaT Cells

The cells were trypsinized, collected, fixed with 70% ethanol and drop-by-drop ice, and stored at −20 °C overnight. Then, the cells are washed with PBS, and annealed with 200 mkg/mL of RNase (PanReac, AppliChem, Germany) and 20 mkg/mL of propidium iodide (Sigma-Aldrich, St. Louis, MO, USA) for 30 min at 37 °C. DNA content was analyzed using a Cell Muse analyzer (Luminex, Austin, TX, USA). The data were processed using Muse 1.5 analytics software (Luminex, Austin, TX, USA). The quantity of cells at each stage of the cell cycle was expressed as a percentage.

### 4.10. Luciferase Measure of the Transcriptional Activity of NF-κB

Experiments were performed as previously described [17]. The viable JB6 Cl41 NF-κB cells (8 × 10^3^ cell/well) were seeded into each well of a 96-well plate for overnight, and then the compounds at a concentration of 1 mM were added for 3, 6, or 24 h.

Then, the cells were disrupted for 1 h at RT with lysis buffer (0.1 M potassium phosphate buffer at pH 7.8, 1% Triton X-100, 1 mM DTT, 2 mM EDTA) and 30 µL of lysate from each well was transferred into a plate for luminescent analysis, and luciferase activity was measured using luciferase assay buffer (100 µL/well) containing 0.47 mM D-luciferin, 20 mM Tricin, 1.07 mM (MgCO_3_)_4_ × Mg(OH)_2_ × 5H_2_O, 2.67 mM MgSO_4_ × 7H_2_O, 33.3 mM DTT, 0.53 mM ATP, 0.27 mM CoA, and 0.1 mM EDTA, pH 7.8. The measurement was performed using a Luminoscan Ascent Type 392 microplate reader (LabSystems, Helsinki, Finland). The results are expressed as the luminescence intensity.

### 4.11. Protein Electrophoresis and Immunoblotting

After treatment with the compounds (10 µM), the cells were washed with PBS (BioloT, Russia) and lysed using radioimmunoprecipitation assay buffer (Sigma-Aldrich, St Louis, MO, USA). The Bradford method was used to measure protein concentration. SDS-PAGE on a 12% polyacrylamide gel was performed to separate the proteins, which were transferred onto an Immobilon^®^-P polyvinylidene difluoride (PVDF) membrane using a semi-dry transfer cell (Bio-Rad, Hercules, CA, USA). Specific primary rabbit antibodies against glutathione synthetase (Cat. NO: A11557, Abclonal, MA, USA) and phospho-NRF2-S40 (Cat. AP1133, Abclonal, MA, USA) were used to detect the protein zones, while specific monoclonal antibodies against β-actin were used to detect the β-actin protein zones as a loading control. Horseradish peroxidase (HRP)-conjugated antibodies were purchased from Sigma-Aldrich, Louis, MO, USA) and secondary antibodies were used. Protein zones on the membrane were visualized using a PierceTM ECL kit (Thermo Fisher Scientific, Waltham, MA, USA) and a Versa Doc imaging system (Bio-Rad, Hercules, CA, USA). Densitometric analysis of the protein zones was performed using the Image Lab 6.0.1 software (Bio-Rad, Hercules, CA, USA).

### 4.12. Molecular Docking

The PDB file of the Kelch domain of Keap1 (PDB ID 1U6D) was obtained from the RCSB Protein Data Bank (https://www.rcsb.org). The PrepDock package of UCFS Chimera 1.16 software was used to prepare the target molecule for docking. The chemical structures of asterripeptides A–C were prepared for docking by ChemOffice and checked using the PrepDock package of UCFS Chimera 1.16 software. Docking was conducted on the SwissDock online server (http://www.swissdock.ch) using EADock DSS docking software [46]. The algorithm involves the generation of a number of binding modes in the vicinity of all target cavities (blind docking) and estimation of their CHARMM energies [47] to evaluate the binding modes with the most favorable energies from the Fast Analytical Continuum Treatment of Solvation (FACTS) [48] and, finally, clustering of these binding modes [49].

The predicted building models for the Kelch/asterripeptide A–C pairs were analyzed using UCFS Chimera 1.16 software. Docking parameters, such as Gibb’s free energy (ΔG, kcal/mol), FullFitness score (FF, kcal/mol), hydrogen bonding (H-bond), and hydrophobic interactions, were used for the analysis of the target/ligand complexes, as described in [18].

### 4.13. In Vivo Experiments

#### 4.13.1. Animals and Treatment

Mature white male BALB/C mice, weighing 20 ± 1.5 g, were used in this study. The animals were kept under standard conditions in accordance with the rules of SP 2.2.1.3218-14 on the device, equipment, and maintenance of experimental biological vivariums and GOST 33216-2014 “Guidelines for the maintenance and care of animals”. The animals were kept under controlled environmental optimal parameters with a temperature of 23 ± 3 °C, humidity of 50%, and 12 h lighting cycle. The mice had constant access to a balanced delta feed, laboratory animal feed, and filtered water. All experimental work with animals was carried out in accordance with the European Directive 2010/63/EC “On the protection of animals used for scientific purposes” and the rules of GOST 33044-2014 “Principles of good laboratory practice”. Work with animals was approved by the local ethics committee of the PIBOC FEB RAS No. 03/24 (17 May 2024).

The mice were divided into five groups, six in each: group I, intact mice for blood control; group II, untreated mice; group III, mice treated with asterripeptide C; group IV, mice treated with the reference drug levomekol; and group V, mice treated with CMC hydrogel without active compound. Before manipulation, animals were anesthetized with a mixture of zoletil and xylazine 20–40 mg/kg + 5–10 mg/kg injection. The experiment was terminated after complete wound healing in the experimental groups and the mice were euthanized.

Asterripeptide C (**3**) was dissolved in small amount of DMSO (100 µL) and then dissolved in carboxymethylcellulose (CMC) hydrogel [21]; for example, the concentration of **3** was 0.5 mkg per g of gel. CMC hydrogel with DMSO without the compound was used as the base control. Levomekol (Nizhfarm, Russia) was used as a reference drug. Thin layers of CMC hydrogel, with or without compounds, were applied to each wound using a sterile spatula.

#### 4.13.2. Burn Wound

To obtain a burn wound model, a thermal burn was applied using a copper rod with a flat end face and a diameter of 0.6 cm. The rod was heated in a boiling water bath and pressed against a shaved area of the skin in the interscapular region for 6 s. Two burns were formed in each mouse. Treatment was initiated a day after the burn was applied.

#### 4.13.3. *S. aureus*-Infected Incised Wound

A skin biopsy device and surgical scissors were used to create the incision wound model. Two wounds were created per mouse. After 2 h, the wounds were infected with *S. aureus* suspension at 0.5 × 10^9^ CFU/mL (10 µL of suspension per each wound) in sterile phosphate buffer saline. Treatment was initiated a day after the burn was applied.

#### 4.13.4. The Measurements and Peripheral Blood Analysis

The wounds were photographed and the area was measured using ImageJ 1.53t software (Rasband W.S., NIH). To assess the dynamics of wound infection, measured crops of 10-fold dilutions of purulent discharge were added on selective media (pancreatic hydrolysate of fish meal (5.0 g/L), peptone (5.0 g/L), pancreatic hydrolysate of caseine (20.0 g/L), yeast extract (2.0 g/L), NaCl (68.0 g/L), mannite (10.0 g/L), phenol red (0.025 g/L), and agar (10.0 ± 3.0 g/L), pH 7.2 ± 0.2) from Scientific Centre of Applied Microbiology and Biotechnology (Obolensk, Russia), and counting of grown colonies (CFU, amount/wound) was carried out. The levels of TNF-α and IL-18 cytokines in the blood of the animals and a clinical blood test were performed four days after the completion of the experiment. Blood samples were collected and centrifuged for cytokine analysis. TNF-α levels were analyzed using the Mouse TNF-α ELISA kit (SEA133Mi), and IL-18 levels were analyzed using the Mouse IL-18 ELISA kit (SEA064Mu) from Cloud-Clone (Houston, TX, USA) in accordance with the manufacturer’s instructions. Clinical blood tests were performed using a hematology analyzer (Mindray BC-5000 Vet, Guangzhou, China).

### 4.14. Statistical Data Evaluation

All data were obtained from three independent replicates, and the calculated values are expressed as the mean ± standard error of the mean (SEM). Student’s *t*-test was performed to determine significant differences using SigmaPlot 14.0 (Systat Software Inc., San Jose, CA, USA).

## 5. Conclusions

Thus, marine fungal asterripeptides A–C at a concentration of 10 µM inhibited *S. aureus* growth and biofilm formation. Asterripeptides A–C increased the viability, proliferation, and migration of *S. aureus*-infected HaCaT cells, as well as reduced the release of reactive oxygen species, NO, TNF-α, and IL-18 from these cells. Moreover, asterripeptides A–C protected HaCaT cells against TNF-α-induced inflammation and decreased the transcriptional level of NF-κB in JB6 Cl41 cells, as well as enhanced the protein levels of Nrf2 and glutathione synthetase in HaCaT cells. More active asterripeptide C was tested in in vivo burn wounds and *S. aureus*-infected incised wounds. Asterripeptide C significantly enhanced wound healing and normalized cytokine levels and profiles of peripheral blood samples, as well as decreased *S. aureus* contamination of wounds and blood in mice with infected incised wounds. Taken together, these results confirm the dual antibacterial and Nrf2-dependent anti-inflammatory activities of asterripeptides A–C in in vitro and in vivo assays.

## Figures and Tables

**Table 1:** The antibacterial activity of asterripeptides A–C (**1**–**3**).

Compound	Growth Inhibition, %			Biofilm Formation Inhibition, %	
	100 µM	50 µM	25 µM	100 µM	10 µM
**1**	57.33 ± 1.12	52.85 ± 2.01	-	52.33 ± 2.06	62.79 ± 1.49
**2**	49.79 ± 0.78	28.96 ± 1.22	-	54.04 ± 4.99	52.63 ± 3.99
**3**	35.30 ± 1.15	34.85 ± 3.40	13.56 ± 1.08	53.79 ± 6.99	61.92 ± 5.79

**Table 2:** The calculated complexes of asterripeptides A–C (**1**–**3**) with Kelch domain of Keap1.

Compound	ΔG, kcal/mol	FF Score, kcal/mol	H-Binding Interactions, Å	Hydrophobic Interactions
**1**	−10.0638	−1084.234	Gly605 … O, 3.709 Gly367 … O, 1.933	Ala556, Ile416, Gly603, Leu365, Ala366, Ile559, Val512, Cys513, Val514, Val561, Thr560, Ala607, Cys368, Val420
**2**	−9.657	−1045.076	Ile559 … O, 2.352	Ala556, Gly364, Gly603, Leu365, Ala336, Leu557, Gly558, Ile559, Val512, Cys513, Thr560, Val561, Val514, Val420, Gly419, Ser486
**3**	−9.429	−1071.740	Ile559 … O, 2.208	Ala556, Gly364, Val604, Gly509, Leu365, Ala510, Leu557, Val465, Ala366, Val418, Val606, Ile559, Gly367, Cys368, Val369, Thr560, Ala466, Cys513, Val514

The underline indicates the amino acid residue formed the enzyme active site.

**Table 3:** The effect of asterripeptide C (**3**) on the peripheral blood profile of mice with burn wounds.

Indicator	Intact	Untreated	3	Base	Levomekol
	Day 18				
WBC, 10^9^/L	6.447 ± 1.526	6.813 ± 1.985	4.750 ± 1.208	4.983 ± 0.818	6.397 ± 1.970
Neu, 10^9^/L	1.703 ± 0.418	3.615 ± 0.526	1.377 ± 0.471	1.483 ± 0.301	2.167 ± 0.669
Lym, 10^9^/L	4.167 ± 0.708	3.597 ± 0.230	3.073 ± 0.681	3.150 ± 0.630	3.847 ± 1.217
Mon, 10^9^/L	0.347 ± 0.121	0.197 ± 0.087	0.147 ± 0.047	0.150 ± 0.044	0.197 ± 0.072
Eos, 10^9^/L	0.230 ± 0.070	0.190 ± 0.075	0.150 ± 0.026	0.197 ± 0.072	0.187 ± 0.042
Bas, 10^9^/L	0.000 ± 0.000	0.013 ± 0.006	0.003 ± 0.006	0.003 ± 0.006	0.000 ± 0.000
RBC, 10^12^/L	9.307 ± 0.341	8.653 ± 1.286	9.620 ± 0.310	9.620 ± 0.495	9.253 ± 0.146
HGB, g/L	148.00 ± 9.54	134.00 ± 28.00	152.33 ± 4.04	152.00 ± 7.00	140.00 ± 2.00
PLT, 10^9^/L	376.00 ± 69.60	549.33 ± 73.71	328.00 ± 16.97	524.00 ± 73.54	468.00 ± 167.10

The data are presented as the mean ± standard error of mean (SEM).

**Table 4:** The effect of asterripeptide C (**3**) on the blood profiles of mice.

Indicator	Intact	Untreated	3	Base	Levomekol
	Day 4				
WBC, 10^9^/L	4.463 ± 1.042	5.820 ± 2.260	4.820 ± 1.000	5.680 ± 0.612	4.557 ± 1.330
Neu, 10^9^/L	1.290 ± 0.365	2.798 ± 0.391	1.953 ± 0.534	2.057 ± 0.283	1.870 ± 0.678
Lym, 10^9^/L	2.773 ± 0.391	2.647 ± 0.615	2.610 ± 0.548	3.243 ± 0.344	2.447 ± 0.621
Mon, 10^9^/L	0.197 ± 0.115	0.277 ± 0.096	0.133 ± 0.029	0.227 ± 0.091	0.130 ± 0.010
Eos, 10^9^/L	0.200 ± 0.176	0.117 ± 0.012	0.120 ± 0.036	0.153 ± 0.012	0.110 ± 0.044
Bas, 10^9^/L	0.003 ± 0.006	0.000 ± 0.000	0.003 ± 0.006	0.000 ± 0.000	0.000 ± 0.000
RBC, 10^12^/L	9.363 ± 0.317	10.143 ± 0.151	9.857 ± 0.446	9.787 ± 0.147	9.363 ± 0.887
HGB, g/L	145.333 ± 7.506	152.667 ± 3.215	155.000 ± 6.083	155.667 ± 1.528	156.000 ± 10.440
PLT, 10^9^/L	377.333 ± 47.606	587.667 ± 34.686	495.333 ± 58.398	554.667 ± 71.699	563.000 ± 136.451
	Day 12				
WBC, 10^9^/L	6.177 ± 1.236	2.520 ± 1.942	4.535 ± 0.932	4.497 ± 0.391	4.397 ± 0.569
Neu, 10^9^/L	2.047 ± 0.684	0.665 ± 0.148	1.370 ± 0.303	1.687 ± 0.505	1.857 ± 0.559
Lym, 10^9^/L	3.697 ± 0.529	1.137 ± 1.078	2.880 ± 0.690	2.477 ± 0.110	2.25 ± 0.335
Mon, 10^9^/L	0.243 ± 0.049	0.170 ± 0.069	0.155 ± 0.029	0.163 ± 0.035	0.137 ± 0.035
Eos, 10^9^/L	0.190 ± 0.010	0.113 ± 0.064	0.130 ± 0.107	0.170 ± 0.050	0.153 ± 0.101
Bas, 10^9^/L	0.000 ± 0.000	0.000 ± 0.000	0.000 ± 0.005	0.000 ± 0.000	0 ± 0
RBC, 10^12^/L	9.060 ± 0.565	5.263 ± 3.846	9.210 ± 1.740	9.227 ± 0.220	8.737 ± 1.154
HGB, g/L	142.667 ± 9.018	86.333 ± 6.517	155.000 ± 24.069	147.333 ± 3.055	137.333 ± 16.773
PLT, 10^9^/L	536.667 ± 104.026	338.667 ± 322.274	563.000 ± 9.899	207.500 ± 19.092	940.333 ± 264.462

## Data Availability

The original contributions presented in the study are included in the article; further inquiries can be directed to the corresponding author.
